# Ultrasound-assisted extraction of bioactive pigments from *Spirulina platensis* in natural deep eutectic solvents

**DOI:** 10.1186/s40643-023-00692-x

**Published:** 2023-12-01

**Authors:** Rodrigo Martins, Cláudia Mouro, Rita Pontes, João Nunes, Isabel Gouveia

**Affiliations:** 1 FibEnTech Research Unit, Faculty of Engineering, University of Beira Interior, 6200-001 Covilhã, Portugal; 2 Association BLC3-Technology and Innovation Campus, Centre Bio R&D Unit, 3405-155 Oliveira do Hospital, Portugal; 3 BLC3 Evolution Lda, 3405-155 Oliveira do Hospital, Portugal

**Keywords:** Green chemistry, Green extraction, Microalgae, Optimal, Bioeconomy

## Abstract

*Spirulina platensis* biopigments have been documented as a potential source of nutritional, physiological, and pharmacological purposes due to the presence of bioactive pigments, total phenolic content (TPC) and the consequent antioxidant activity that these compounds present. Bioextracts market has increased in the last decades and is a key option for replacing fossil-derived products and promote the transition for a bio-based economy. To take advantage of these compounds more effectively, optimized extraction processes must be researched and used in biomass sources. The present study focused on optimizing the ultrasound-assisted extraction (UAE) using response surface methodology. Three factor and three level Box–Behnken design was used to optimize the extraction of bioactive pigments, and to investigate the effects of three independent variables, $x$_1_: extraction temperature (40–60 °C), $x$_2_: extraction cycle time (20–40 min), and $x$_3_: solvent-to-biomass ratio (50–70 mL/mg) on total pigment yield, antioxidant assay, and TPC (dependent variables). A second-order polynomial model was used for predicting the responses. Statistically, the model was validated using an analysis of variance. Results revealed that ultrasound-assisted temperature, time, and solvent-to-biomass ratio had a significant (*p* < 0.05) influence on the total pigment yield, while temperature and solvent-to-biomass ratio had a significant influence in the antioxidant activity, and temperature significantly influenced the total pigment yield. For total pigment yield, antioxidant activity, and total phenol content, the ${R}^{2}$ values of the models generated were 0.8627, 0.8460, and 0.9003, respectively, indicating that the models developed based on second-order polynomials were satisfactorily accurate for analyzing interactions between parameters. Desirability functions showed that the optimal extraction parameters were temperature: 60 °C, extraction cycle time: 20 min; and a solvent-to-biomass ratio of 70 mL/mg. Under optimal conditions, experimental values for total pigment yield, total phenol content expressed as gallic acid equivalent (GAE), and antioxidant activity expressed as Trolox equivalent (TRE) were: 165.19 ± 1.01 mg/g Dry Matter (DM), 36.50 ± 0.98 mg GAE/g DM, and 37.98 ± 0.58 mg TRE/g DM, respectively. The experimental values showed a good agreement with the predicted values with residual standard low 1% under optimum conditions. This optimized ultrasound-assisted method in natural eutectic solvents is effective and scalable to a green extraction of the bioactive pigments from *Spirulina platensis* with potential application to food, pharmaceutical, functional materials, and packaging.

**Graphical Abstract:**

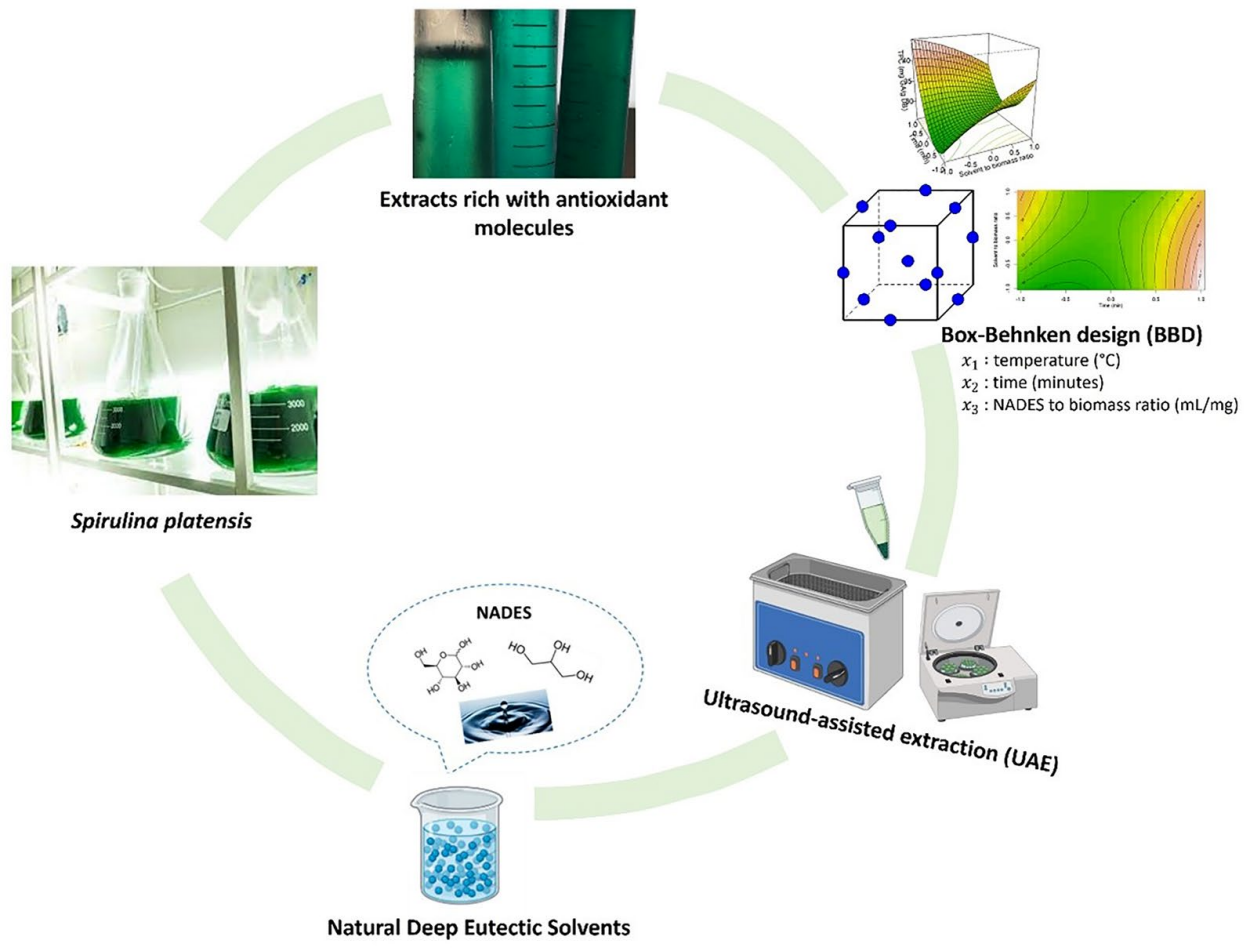

## Introduction

Bioactive pigments are interesting compounds present in several microalgae species that have drawn much attention from researchers and society at large, mainly due to their unique bioactive properties, color, and possible applications. Due to the ambitious sustainable goals set by the United Nations, which aim at reducing the dependency of the world’s economy on synthetic materials, natural bioactive pigments are becoming more relevant in society (Martins et al. 2023a, b). Several bioactive pigments have already been used for several applications mainly in the pharmaceutical and packaging industries (Kholany et al. 2022; Moreira et al. 2018). Pigments such as anthocyanins and phycocyanin have been used in the development of pH-sensitive packaging, where the changes of color with pH serves as an indicator of food freshness (Bhargava et al. 2020; Moreira et al. 2018). Moreover, has shown by our research group (Martins et al. 2023a), these bioactive phytochemicals can also be incorporated into materials using techniques such as electrospinning and co-polymers such as polyvinyl alcohol (PVA).

To increase productivity, decrease processing time, and conserve energy, the use of green extraction processes is quite relevant. Ultrasound-assisted extraction (UAE) is becoming more popular as an alternative technology, since it has been utilized to reduce process temperature, time, and solvent use (Ahmed et al. 2022).

In a UAE, ultrasonic waves induce a process known as cavitation, which results in a rapid series of compression and expansion waves near the surface of a solid matrix. Decompression causes giant air bubbles to grow, eventually collapsing and imploding, releasing the stored energy as waves. The microscopic channels formed by the procedure above create a sponge effect in tissues, allowing the solvent to penetrate and release the compounds of interest more easily (Ahmed et al. 2022).

Coupled with extraction techniques, different extraction solvents are often used in extraction processes. The recovery of high value bioactive compounds from several natural sources—including microalgae—is largely realized using traditional organic solvents. Nevertheless, these solvents are often related to low yield efficiency and increased energy consumption, as well as toxicity, volatility, flammability, non-biodegradability, and non-renewability. Given these drawbacks and considering the principles of green chemistry, new alternative solvents have been introduced for the extraction of bioactive compounds, such as ionic liquids (ILs), deep eutectic solvents (DES), and natural deep eutectic solvents (NADES) (Martins et al. 2023a, b).

The extraction efficiency of bioactive pigments, namely, antioxidant activity, and total phenolic content (TPC) can be affected by ultrasonic extraction cycles, ultrasonic duration, ultrasonic temperature, solvent concentration, solvent acidity, and solvent type employed in the UAE. Considering the UAE of microalgae species, several authors have investigated the extraction of bioactive compounds from microalgae species based on the UAE technique and suggested three key factors affecting extract composition are ultrasonic time, ultrasonic temperature, and solvent used (Ahmed et al. 2022).

For optimizing analytical procedures, Response Surface Methodology (RSM) is frequently used in the several fields. RSM is less time consuming and less labor intensive than others, since it requires, due to the fewer experimental trials needed to analyze the effects of multiple extraction parameters and their interactions. Many experiments have utilized the RSM's most frequent designs, such as central composite design (CCD) and Box–Behnken design (BBD). BBD for the RSM is explicitly developed to fit a second-order model, the primary focus of most RSM investigations. Besides, the BBD only requires three levels of each factor to fit a second-order regression model, whereas CCD requires five levels for each factor. In addition, BBD takes fewer experimental runs (Lenth 2020).

In this study, *Spirulina platensis* was submitted to UAE using a glucose/glycerol and water-based NADES, forming a gel-like mixture with great antioxidant potential. UAE was conducted varying extraction parameters, such as temperature, time of extraction, and solvent-to-biomass ratio, in three different levels for each variable. Then, the gel-like extracts were submitted to three assays: pigment determination through spectrophotometry, antioxidant activity determination through the ABTS assay, and TPC determination through the Folin–Ciocalteu's method. The response variables were fitted to second-order polynomial models. Thereafter, desirability functions were used to determine the optimal conditions for the extraction process. Finally, more experimental runs were done to verify the model’s appropriateness.

## Materials and methods

### Chemicals

D-glucose anhydrous (MW = 180.16 g mol^−1^), glycerol (MW = 92.09 g mol^−1^), and acetic acid glacial (MW = 60.052 g mol^−1^) were purchased from Fisher Scientific (Hampton, NH, USA). ABTS (MW = 548.69 g mol^−1^), potassium peroxodisulphate (MW = 270.32 g mol^−1^), sodium acetate anhydrous (MW = 82.03 g mol^−1^), and Folin–Ciocalteu's reagent (MW = 260.20 g mol^−1^) were purchased from PanReac (Barcelona, Spain). Trolox (MW = 250.29 g mol^−1^) was purchased from ThermoScientific (Waltham, Massachusetts, USA). Gallic acid monohydrate (MW = 188.12 g mol^−1^) was purchased from AcrosOrganics (Geel, Belgium). Sodium carbonate anhydrous (MW = 105.99 g mol^−1^) was purchased from LabChem (Zelienople, Pennsylvania, US).

### Biomass production: *Spirulina platensis*

*Spirulina platensis* was maintained in Zarrouk culture medium in a thermostatic greenhouse at 25 ± 2 °C. The agitation of the culture and the supply of CO_2_ was carried out through the continuous injection of air. The light intensity was maintained at 12 µmol s^−1^ m^−2^ and the photoperiod 12 h light and 12 h dark. *S. platensis* biomass was then obtained through vacuum filtration. Afterwards, the biomass was freeze-dried and kept in polypropylene flasks until further use.

### Green extraction: ultrasound-assisted extraction (UAE) using natural deep eutectic solvents (NADESs)

UAE was carried out using an ultrasonic bath (Labbox, Barcelona, Spain) with a capacity of 10 L, 240 W, and 40 kHz, and a glucose/glycerol/water-based (1:2:4 molar ratio) NADES. The UAE was adapted from Wils et al. (Wils et al. 2021) with minor modifications. Box–Behnken experimental design (BBD) (Table 1) in a three-level three-factor full factorial design with three center points was used. Three independent variables were identified as ${x}_{1}$ (temperature in °C), ${x}_{2}$ (time in minutes), ${x}_{3}$ (solvent-to-biomass ratio in mL/mg).

**Table 1:** Box–Box–Behnken design (BBD) used in this research article

	Independent variables			Coded independent variables		
Run	Temperature (°C) × 1	Extraction cycle time (min) × 2	Solvent-to-biomass ratio (mL/mg) × 3	Temperature (°C) × 1	Extraction cycle time (min) × 2	Solvent-to-biomass ratio (mL/mg) × 3
1	50	20	60	− 1	− 1	0
2	70	20	60	1	− 1	0
3	50	30	60	− 1	1	0
4	70	30	60	1	1	0
5	50	30	50	− 1	0	− 1
6	70	30	50	1	0	− 1
7	50	30	70	− 1	0	1
8	70	30	70	1	0	1
9	60	20	50	0	− 1	− 1
10	60	40	50	0	1	− 1
11	60	20	70	0	− 1	1
12	60	40	70	0	1	1
13	60	30	60	0	0	0
14	60	30	60	0	0	0
15	60	30	60	0	0	0

### Spectrophotometry analysis of the *Spirulina platensis* extracts

Spectrophotometry analysis was conducted in UV5, Mettler Toledo. For the analysis, the aliquots were filled with the extracts, then the highest absorbance at a specific wavelength spectrum was determined, and the concentration of bioactive pigments such as chlorophylls a and b, carotenoids, and phycocyanin were determined using Eqs. 1, 2, and 3, respectively (Aderemi 2020; Yang et al. 1998):

1 $$\begin{array}{c}{C}_{chlorophyll\mathrm{ a}+b} \left(\mu g {ml}^{-1}\right) =17.76{A}_{646.6}-7.34{A}_{663.6}\end{array}$$

2 $$\begin{array}{c}{C}_{carotenoids} \left(\mu g {ml}^{-1}\right) =4.69{A}_{440}-0.267 {C}_{chlorophyll\mathrm{ a}+b}\end{array}$$

3 $$\begin{array}{c}{C}_{phycocyanin} \left(mg {ml}^{-1}\right)= {(A}_{620}-0.474{A}_{652})/5.34\end{array}$$

where C_chlorophyll a+b_ is the concentration of chlorophyll a and b in μg/ml, C_carotenoids_ is the concentration of total carotenoids in μg/ml, and C_phycocyanin_ is the concentration of phycocyanin in mg/ml. In addition, A_663.6_, A_646.6_, A_440_, A_620_ and A_652_ are the absorbance values at 663.6, 646.6, 440, 620 and 652 nm, respectively.

The yield of each pigment was determined following equation, also used by other authors (Sutanto & Suzery 2015):

4 $$\begin{array}{c}Yield \left(\frac{mg}{g}\right)= {C}_{pigment}\times \frac{V}{DB}\end{array}$$

where C_pigment_ is the concentration of chlorophyll a and b, carotenoids, and phycocyanin expressed in mg/mL, V is the volume of solvent expressed in mL, and DB is the dried biomass used in the extraction, expressed in g.

### Determination of bioactive properties

#### Determination of antioxidant activity through ABTS assay

Five milliliters of ABTS solution (7 mmol/L) and 5 ml of potassium persulfate solution (2.45 mmol/L) were mixed and kept at room temperature for 12–16 h in dark to make ABTS reaction solution. ABTS reaction solution of 2.80 mL was diluted to 65 mL in acetate buffer with pH 4.5 to obtain ABTS working solution, and kept at room temperature for 30 min in dark. The absorbance at 734 nm was 0.72 ± 0.02 (Xiao et al. 2020).

Then, in a 96-well plate, 200.0 µL ABTS working solution and 10.0 µL sample solution of different concentrations were added, shook well, and protected from light for 7 min. The absorbance was measured at 734 nm. Trolox was used as the standard and distilled water as the blank control. Trolox concentration was selected under the condition of absorbance value ranging from 0.2 to 0.8 to draw a standard curve (${R}^{2}$=0.96). In this assay, the extract or sample solution is also diluted to an absorbance value in the range from 0.2 to 0.8 (Xiao et al. 2020).

The result was expressed as µg Trolox equivalent (TRE)/g DM (Dry Matter) according to the following equation:

5 $$\begin{array}{c}ABTS \left(\upmu g\frac{TRE}{g}DM\right)=C\times V\times \frac{t}{m}\end{array}$$

where $C$ is the Trolox concentration (µg/mL) of the corresponding standard curve of the diluted sample, $V$ is the sample volume (mL), $t$ is the dilution factor, and $m$ is the weight of the sample dry matter (g).

#### Determination of total phenolic content (TPC)

To determine the TPC of *Spirulina platensis* extracts the Folin–Ciocalteau method was used. Thus, *Spirulina platensis* extracts (40 μL) were added together with 80 μL of Folin–Ciocalteau reagent 10% (m/v) and 100 μL sodium carbonate solution at a concentration of 7.5% (m/v) in a multi-well polystyrene plate. The solution was incubated at dark ambient for 2 h. The absorbance was determined using the spectrophotometer (UV5, Mettler Toledo, USA) at 750 nm. The “mg GAE (gallic acid equivalents)/100 g DM (Dry Matter) of sample” was used to express the results using a gallic acid standard curve (${R}^{2}$ = 0.97).

### Experimental design and statistical analysis

The present study used a BBD in the form of a three-level three-factor full factorial design for each independent variable to evaluate the effects of process variables associated with the UAE on the response variables. Three process variables were selected: ${x}_{1}$ (temperature: 50–70 °C), ${x}_{2}$ (time: 20–40 min), and ${x}_{3}$ (solvent-to-biomass ratio: 50–70 mL/mg) and a total of 15 runs were performed (Table 1). After obtaining the results for each dependent variables (total pigment yield, antioxidant activity, and TPC), RSM was used to determine the optimal processing settings for each of the three independent variables. The influence of temperature, time, and solvent-to-biomass ratio on bioactive pigment yield, antioxidant capacity, and TPC, were investigated using a second-order polynomial equation [Eq. (6)] obtained from RSM:

6 $$\begin{array}{c}y = {a}_{0}+{a}_{1}{x}_{1}+{a}_{2}{x}_{2}+{a}_{3}{x}_{3}+{a}_{12}{x}_{1}{x}_{2}\quad+{a}_{13}{x}_{1}{x}_{3}+{a}_{23}{x}_{2}{x}_{3}+{a}_{11}{x}_{1}^{2}+{a}_{22}{x}_{2}^{2}+{a}_{33}{x}_{3}^{2}\end{array}$$

where y is the measured response variables, ${x}_{1}$, ${x}_{2}$, and ${x}_{3}$ represent the independent variables, ${a}_{0}$ is a constant (predicted response at the center), ${a}_{1}$, ${a}_{2}$, ${a}_{3}$; ${a}_{11}$, ${a}_{22}$, ${a}_{33}$; and ${a}_{12}$, ${a}_{13}$, ${a}_{23}$, are the linear, quadratic, and two-factor interaction coefficients of the model, respectively. The RStudio® (Version 2022.12.0) statistical software was used for the experimental design and the analysis of variance (ANOVA) to determine the effects of significant interactions in the model (*p* < 0.05).

Pearson correlation test was also employed at a *p* value of 0.05, to determine significant correlations between total pigment yield, TPC and antioxidant activity.

### Desirability function analysis (DFA)

Desirability function analysis is a prominent technique applied in academia and industrial arena for simultaneous optimization of multiple responses. In DFA methodology, a process is unacceptable if any one of the responses is outside some desired limits. Thus, individual desirability functions are used based on the objective of the study: maximizing a response; minimizing a response; or reach a target value.

In the case of this research work, all of the response variables were maximized. Thus, the individual desirability functions were calculated based on the following equation:

7 $$  d_{i}  = \left\{ {\begin{array}{*{20}c}    {0,}  \\    {\left( {\frac{{y_{i}  - y_{{\min }} }}{{y_{{\max }}  - y_{{\min }} }}} \right),}  \\    {1,}  \\   \end{array} \begin{array}{*{20}c}    {y_{i}  < y_{{\min }} }  \\    {y_{{\min }}  < y_{i}  < y_{{\max }} }  \\    {y_{i}  < y_{{\max }} }  \\   \end{array} } \right. $$

where ${d}_{i}$ is the individual desirability of each response variable, ${y}_{min}$ is the minimum value accepted of the response variable, ${y}_{max}$ is the maximum value of the response variable, and ${y}_{i}$ is the response of each experimental run (e.g., ${d}_{1}$ is the individual desirability of total pigment yield, ${d}_{2}$ is the individual desirability of the TPC, and ${d}_{3}$ is the individual desirability of the antioxidant activity (ABTS)).

The composite desirability was then determined using the following equation:

8 $$\begin{array}{c}D = {\left({d}_{1}\times {d}_{2}\times {d}_{3}\right)}^\frac{1}{3}\end{array}$$

where *D* is the composite desirability, ${d}_{1}$ is the individual desirability of total pigment yield, ${d}_{2}$ is the individual desirability of the TPC, and ${d}_{3}$ is the individual desirability of the antioxidant activity (ABTS) previously obtained from Eq. (7).

## Results and discussion

### Fitting the model and analysis of variance

**Fig. 1:**
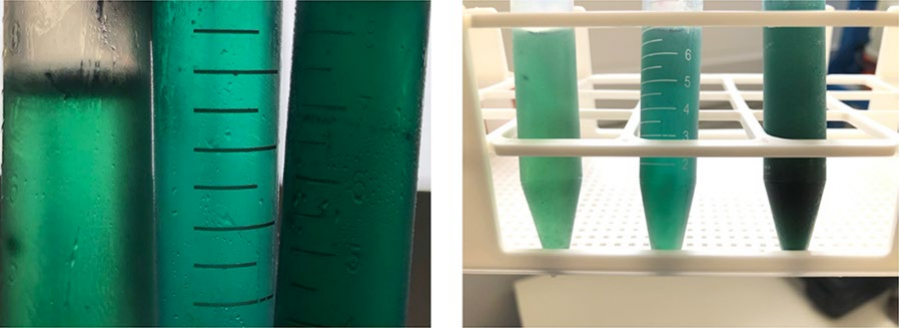
NADES/Spirulina extracts

Using RSM with BBD, the present study examined the effects of UAE time, temperature, and solvent-to-biomass ratio on the total pigment yield, ABTS radical scavenging activity, and TPC of the NADES/Spirulina extracts (Fig. 1). Table 1 summarizes each run conditions from the BBD used in this article.

According to the experimental results (Table 2), total pigment yield, ABTS scavenging activity expressed as 6-hydroxy-2,5,7,8-tetramethylchroman-2-carboxylic acid (Trolox) equivalent (TRE), and TPC expressed as Gallic Acid equivalent (GAE) ranged between: 18.50–161.04 mg/g DM, 21.27–35.36 mg TRE/g DM, and 4.72–43.49 mg GAE/100 g DM, respectively. In addition, experiment 6 (70 °C, 30 min, solvent-to-biomass ratio of 50 mL/mg) provided the lowest pigment yield (18.50 mg/g DM), and the experiment 11 (60 °C, 20 min, solvent-to-biomass ratio of 70 mL/mg) produced the highest pigment yield (161.04 mg/g DM). On the other hand, the NADES/Spirulina extract of experiment 2 (70 °C, 20 min, solvent-to-biomass ratio of 60 mL/mg) showed the highest antioxidant activity (35.36 mg TRE/g DM), and experiment 5 (50 °C, 30 min, solvent-to-biomass ratio of 50 mL/mg) showed the lowest antioxidant activity (21.27 mg TRE/g DM). Moreover, experiment 8 (70 °C, 30 min, solvent-to-biomass ratio of 70 mL/mg) exhibited the lowest phenolic content (4.72 mg GAE/g DM) and experiment 11 (60 °C, 20 min, solvent-to-biomass ratio of 70 mL/mg) showed the highest phenolic content (43.49 mg GAE/g DM).

**Table 2:** Experimental results obtained from the BBD experimental runs

Run	Total pigment yield (mg/g DM)	ABTS (mg TRE/g DM)	TPC (mg GAE/g DM)
1	65.88	27.41	23.15
2	63.37	35.36	7.17
3	66.77	26.93	23.70
4	39.84	30.48	21.98
5	30.08	21.27	14.60
6	18.50	28.92	12.35
7	57.35	27.67	5.73
8	39.92	34.49	4.72
9	64.81	21.73	22.39
10	106.31	25.82	39.99
11	161.04	34.40	43.49
12	83.87	35.05	41.82
13	94.95	27.14	27.97
14	25.10	29.93	31.14
15	60.57	31.25	29.49

Given the results, multiple regression analysis of the actual data resulted in the following second-order polynomial equations for each of the three responses, as illustrated in Eqs. (9), (10), and (11):

9 $$\begin{array}{c}Total\,pigment\,yield \left(\frac{mg}{g}DM\right) =60.2-7.3{x}_{1}-7.3{x}_{2}+15.3{x}_{3}-6.1{x}_{1}{x}_{2}-1.5{x}_{1}{x}_{3}-29.7{x}_{2}{x}_{3}\\ -34.4{x}_{1}^{2}+33.15{x}_{2}^{2}+10.65{x}_{3}^{2} \\ \end{array}$$

10 $$\begin{array}{c}ABTS \left(mg\frac{TRE}{g}DM\right) =29.44-3.24{x}_{1}-0.078{x}_{2}+4.23{x}_{3}-0.28{x}_{1}^{2}+0.89{x}_{2}^{2}+1.07{x}_{3}^{2}\end{array}$$

11 $$\begin{array}{c}TPC \left(mg\frac{GAE}{g}DM\right)= 29.54-2.62{x}_{1}+3.91{x}_{2}+0.80{x}_{3}+3.56{x}_{1}{x}_{2}+0.31{x}_{1}{x}_{3}\\ -4.81{x}_{2}{x}_{3}-19.05{x}_{1}^{2}+8.51{x}_{2}^{2}-1.13{x}_{3}^{2}\end{array}$$

In general, coefficients with a positive sign in the fitted model imply that the variable can enhance the response. In contrast, a negative sign suggests a variable's ability to lower the response. Furthermore, the value of the coefficients indicates the higher or lower relationship that exists between the response variable and the independent variable.

From the equations obtained in the present study [Eqs. (9), (10), and (11)], it was possible to retain several important relationships between the response variables and the independent variables. For instance, from Eq. (9), it was possible to detect that the solvent-to-biomass ratio positively affected the total pigment yield. This was also the case for the antioxidant activity [Eq. (10)], and for the TPC [Eq. (11)]. In the case of binary interactions (${x}_{1}{x}_{2}$, ${x}_{1}{x}_{3}$, and ${x}_{2}{x}_{3}$), the interaction between UAE time and temperature (${x}_{1}{x}_{2}$), and the interaction between the UAE temperature and solvent-to-biomass ratio (${x}_{1}{x}_{3}$), showed positive relations towards the TPC [Eq. (11)].

In the case of ABTS assay [Eq. (10)], UAE temperature ($${x}_{1}$$) and time ($${x}_{2}$$) showed negative effects on the antioxidant activity of the extracts, while the solvent-to-biomass ratio ($${x}_{3}$$) showed positive correlations, indicating an antagonistic effect between the three variables. Furthermore, the quadratic interaction of the extraction time ($${x}_{2}^{2}$$) and solvent-to-biomass ratio ($${x}_{3}^{2})$$ variables positively affected the total pigment yield and the antioxidant activity.

TPC showed positive correlations with extraction time (${x}_{2}$) and solvent-to-biomass ratio (${x}_{3}$). In addition, the quadratic interactions between extraction time variable (${x}_{2}^{2}$) were the only positive quadratic interactions for the TPC model [Eq. (11)], and also this interaction was positive for the total pigment yield [Eq. (9)], and antioxidant activity [Eq. (10)] models. These findings show that the NADES/UAE of *Spirulina platensis* is affected by extraction temperature, time, and solvent-to-biomass ratio in different ways.

Table 3 summarizes the ANOVA analysis of variance for the three models of this research article.

**Table 3:** Analysis of variance (ANOVA) of the quadratic models for total pigments, antioxidant activity and total phenolic content (TPC)

Variable	Total pigment yield (mg/g DM)	ABTS (mg TRE/g DM)	TPC (mg GAE/g DM)
${x}_{1}$	0.4012	0.0042*	0.3199
${x}_{2}$	0.4023	0.9261	0.1604
${x}_{3}$	0.1127	0.0009*	0.7488
${x}_{1}{x}_{2}$	0.6112	–	0.3370
${x}_{1}{x}_{3}$	0.9018	–	0.9303
${x}_{2}{x}_{3}$	0.0464*	–	0.2111
${x}_{1}^{2}$	0.0325*	0.8238	0.0028*
${x}_{2}^{2}$	0.0368*	0.4826	0.0589
${x}_{3}^{2}$	0.4055	0.3996	0.7590
${R}^{2}$	0.8627	0.8460	0.9003
Adjusted ${R}^{2}$	0.6157	0.7305	0.7209
*F* value	3.492	7.3240	5.019
Lack of fit	0.9921	0.4984	0.0332

*Significant data for *p* value < 0.05

Table 3 shows the significance of each variable for the three polynomial models obtained. Several models were first tested before arriving at the models presented in Eqs. (9), (10), and (11). The models presented in this article were chosen based on the best ${R}^{2}$ and adjusted ${R}^{2}$ values, as well as the lowest lack of fit.

Hence, it can be drawn that the two-way interaction between the extraction time and solvent-to-biomass ratio (${x}_{2}{x}_{3}$) was significant (for *p* value of 0.05) for the total pigment yield. In addition, quadratic interactions of extraction temperature (${x}_{1}^{2}$) and extraction time (${x}_{2}^{2}$) also proved to be significant for the model.

Antioxidant activity showed higher adjusted ${R}^{2}$ without the two-way interaction variables (${x}_{1}{x}_{2}$, ${x}_{1}{x}_{3}$, and ${x}_{2}{x}_{3}$). Thus, first-order polynomial and quadratic variables were used to formulate the model. UAE temperature (${x}_{1}$) and solvent-to-biomass ratio (${x}_{3}$) proved to be significant to the model at a *p* value of 0.05.

TPC model proved to have the best adjusted ${R}^{2}$ and the least significant lack of fit, out of the three models obtained. The quadratic term for the extraction time (${x}_{1}^{2}$) proved to be significant (*p* value = 0.05). Since the ${R}^{2}$ values greater than 0.9 reflect an accurately predicted response variable, the models obtained for total pigment yield and antioxidant activity don´t seem to be accurately adjusted. TPC model was the only model that showed a ${R}^{2}$ value greater than 0.9.

Nevertheless, with the BBD and RSM it was possible to obtain second-order polynomial quadratic models that shed light on the effects of UAE variables (i.e., time and temperature) as well as a process variable (i.e., solvent-to-biomass ratio), on the total pigment yield, TPC, and antioxidant activities of NADES and *Spirulina* extracts. With these models it was possible to detect which variables are positively or negatively related to the NADES/UAE of bioactive pigments and phenolic compounds and their consequent antioxidant activity.

### Response surface analysis of total pigment yield

Bioactive pigments extracted from *Spirulina platensis* have shown great properties, such as antioxidant, antimicrobial, and immunomodulatory (Bortolini et al. 2022; Martins et al. 2023a; Martins et al. 2023a, b). Therefore, understanding which levels of the extraction variables positively and negatively affect the total pigment yield of the extraction is quite relevant, since it enhances or inhibits the overall potential of the extraction process and, consequently of the extracts.

Wils et al. (Wils et al. 2021), presented several NADESs formulations that due to their lower and higher solvent polarity, extracted different pigments at different yields. For instance, a menthol and lactic acid-based solvent was able to extract more chlorophylls and carotenoids (lipid soluble pigments) than phycocyanin (water soluble pigment). While a glucose/glycerol/water-based NADES was able to extract both water soluble and lipid soluble pigments at high yields. Although the study presented the first screening of NADESs in UAE of *Spirulina platensis*, optimization of the extraction was missing to reach the best overall pigment yield and other antioxidant properties. In addition, as reported by Chaiklahan et al. (Chaiklahan et al. 2012), glucose might also help stabilize phycocyanin and improve its half-life, helping its use in various applications, such as packaging and pharmaceutical agent.

**Fig. 2:**
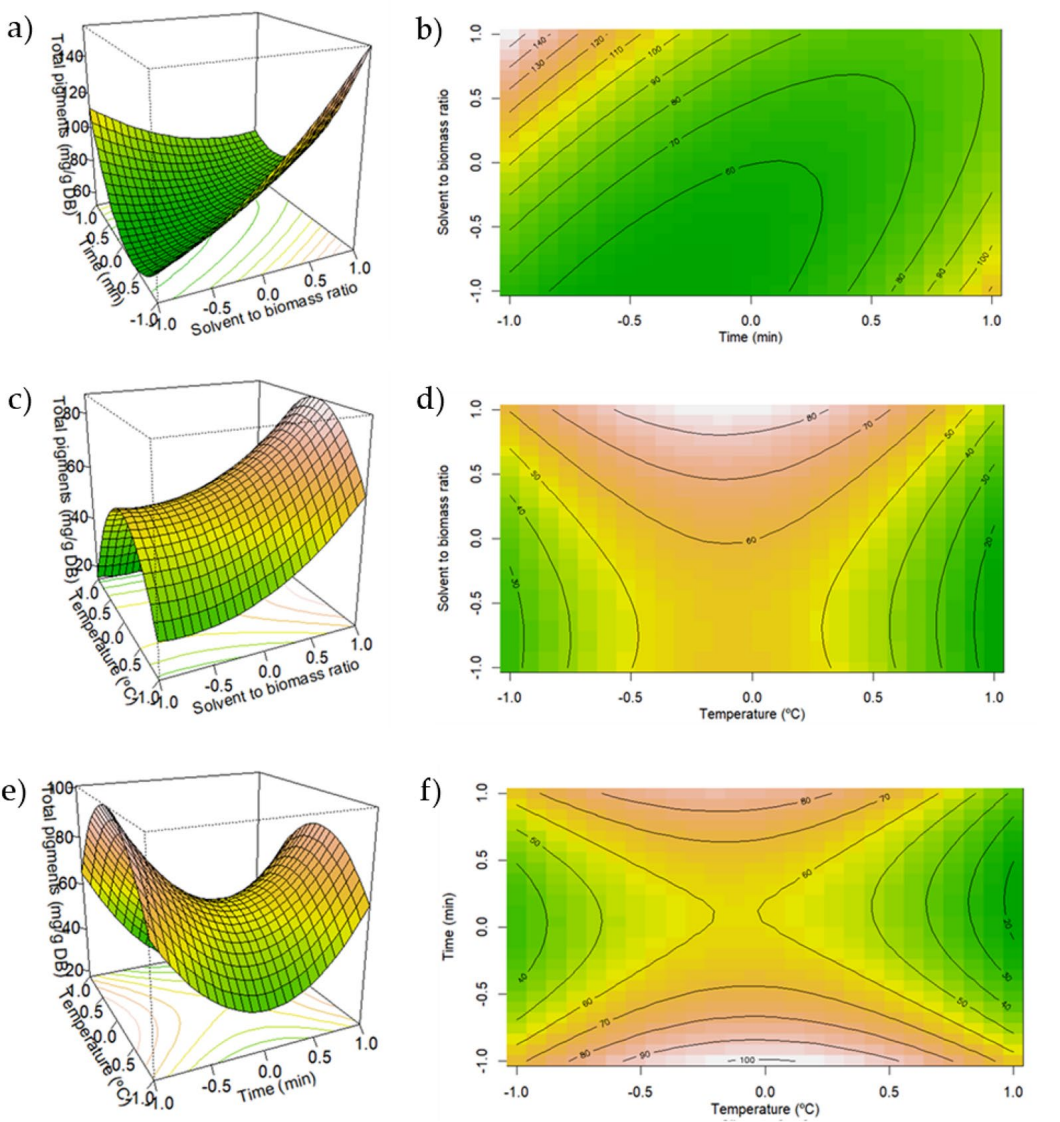
Response surface and contour plots representative of the interaction effects of (**a**, **b**) time and solvent-to-biomass ratio; (**c**, **d**) temperature and solvent-to-biomass ratio; (**e**, **f**) time and temperature on the total pigment yield

In this study, a glucose/glycerol/water-based NADES was used, and the total pigment yield of the NADES/UAE was optimized through RSM. The response surface plot was used to visualize the main effects and interaction effects of the variables used in pigment extraction from the *Spirulina platensis*. The contour plot takes the two variables concurrently while keeping the other variable constant. Figure 2a–f illustrates the response surface plots and the contour plots obtained for the regression model [Eq. (9)].

From Fig. 2a, b, it can be drawn that pigment yield rises with higher solvent-to-biomass ratio and lower time of extraction. Since the solvent induces higher mass transfer from inside the microalgae matrix to the solvent, it takes less time to obtain higher pigment yield (Dardavila et al. 2023).

Figure 2c, d presents similar relations, since a higher solvent-to-biomass ratio showed higher pigment yields. This is in line with other studies that reported the effect of the solvent-to-biomass ratio as a main extraction parameter in MAE and UAE of food wastes and microalgae (Dardavila et al. 2023; Rezaei et al. 2013). Moreover, in a different study focused on the optimization of phycocyanin extraction from *Spirulina platensis* using sodium phosphate buffer as a solvent and maceration as the extraction technique, showed significant relations between solvent-to-biomass ratio and the phycocyanin yield (Silveira et al. 2007).

Time and temperature interactions (Fig. 2e, f) showed that lower time of extraction (three 20 min cycles) and temperature of 60 °C showed higher pigment yields. These extraction times are far shorter than those reported by Dardavila et al. (Dardavila et al. 2023) for the extraction of bioactive compounds from *Chlorella vulgaris* using DES which used times of extraction ranging from 3 to 24 h and similar temperatures of 30 to 60 °C. In addition, despite higher temperatures aid in mass transfer phenomena it can also have an adverse effect on the degradation of the bioactive pigments. Thus, temperatures of above 60 °C don´t show higher pigment yields.

Therewith, for a set temperature, a higher solvent-to-biomass ratio seems to result in higher pigment yields in less extraction time. Moreover, for a fixed solvent-to-biomass ratio, shorter extraction times and temperature of 60 °C show higher pigment yields.

### Response surface analysis of total phenolic content (TPC)

Phenolic compounds or polyphenols are phytochemicals found in most natural matrixes, including food wastes and microalgae (de la Rosa et al. 2019). Phenolic compounds possess numerous bioactive properties, and although they are not nutrients, their dietary intake provides health-protective effects (i.e., antioxidant). Therefore, they are interesting compounds for the pharmaceutical sector (Haoujar et al. 2019).

In a study developed by Alshuniaber et al. 2021, polyphenolic compounds present in *Spirulina platensis* were extracted using methanol and then submitted to GC–MS. Compounds, such as carbonylic acid, phenylacridine, piperidine and others, were found in the methanolic extracts. This study indicated the presence of such phenolic compounds in *Spirulina platensis* extracts. In addition, these compounds showed antimicrobial activity, which is valuable in several applications (i.e., textile, pharmaceutical, etc.).

**Fig. 3:**
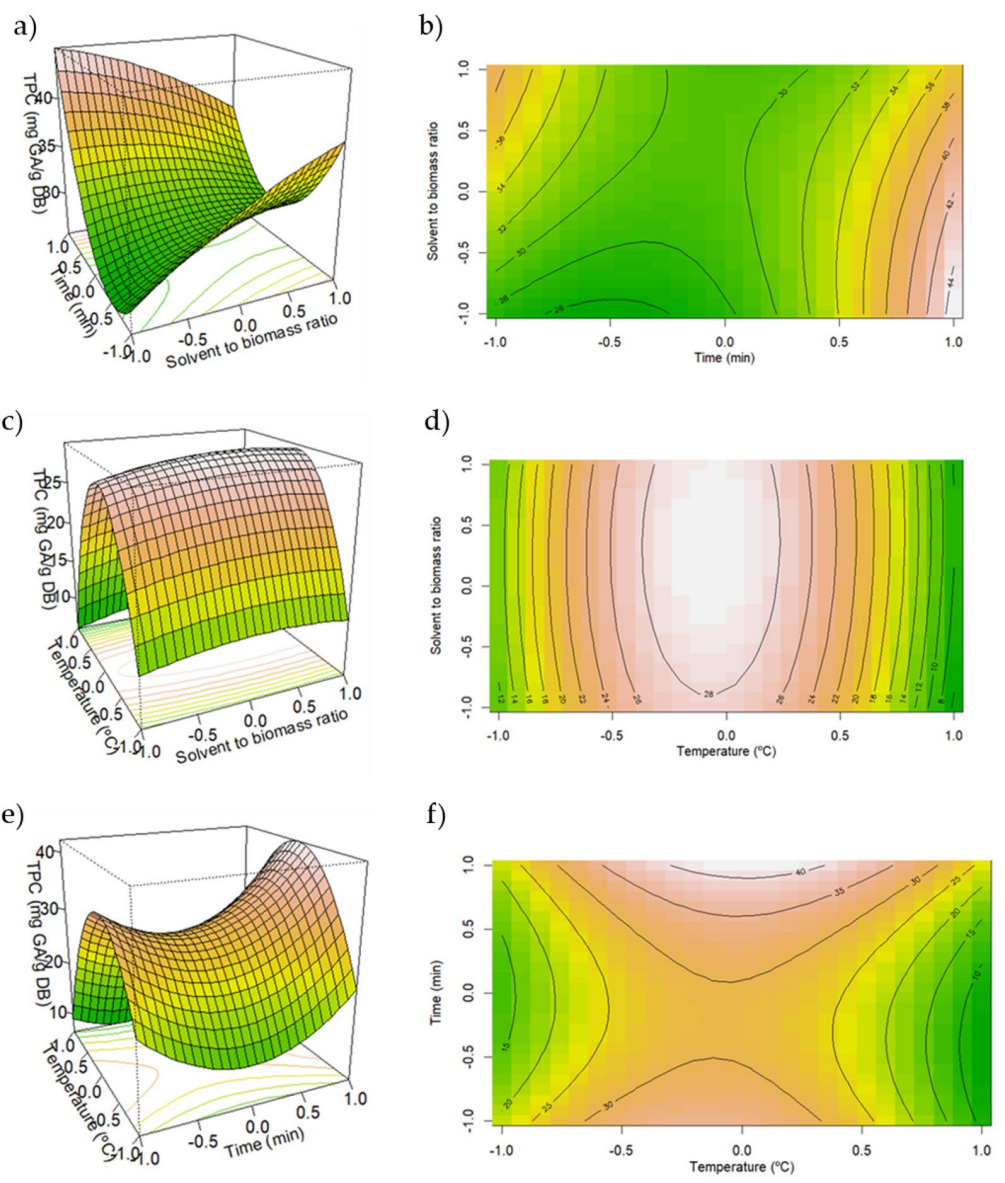
Response surface and contour plots representative of the interaction effects of (**a**, **b**) time and solvent-to-biomass ratio; (**c**, **d**) temperature and solvent-to-biomass ratio; (**e**, **f**) time and temperature on the total phenolic content (TPC)

From Fig. 3a, b, a clear indication of rising TPC with higher time of extraction and higher solvent-to-biomass ratio can be seen. For an extraction process to be the most effective possible, diffusion of the target biomolecules from the biomass to the solvent has to occur (Dardavila et al. 2023). Thus, higher solvent-to-biomass ratio and higher extraction time should aid in the extraction of the target bioactive molecules. Nonetheless, extraction time might have a bigger impact in the extraction process, since, as shown in Fig. 3a, b, the TPC seems to rise again at lower solvent-to-biomass ratios and high extraction times.

For a set time (Fig. 3c, d), a clear indication of higher phenolic content is seen at a temperature of around 60 °C for all ranges of solvent-to-biomass ratio used in this article. In addition, temperatures higher than 60 °C might be related to degradation of phenolics present in the NADES/Spirulina extracts. While temperatures lower than 60 °C don´t insure the correct mass transfer mechanisms necessary for the extraction of the bioactive compounds (i.e., phenolic compounds) from the biomass matrix to the solvent (Dardavila et al. 2023).

For a set solvent-to-biomass ratio (Fig. 3e, f), higher time of extraction yielded the highest phenolic content for temperature of ≈ 60 °C. This is in line with other research works (Dardavila et al. 2023). Therefore, optimization of phenolic compounds through UAE of *Spirulina platensis* seems to be quite dependent on the time and temperature conditions used in the UAE. More precisely, temperatures of ≈ 60 °C and extraction cycle time of 40 min seems to aid in the phenolic content of the NADES/Spirulina extracts.

### Response surface analysis of antioxidant activity

Both bioactive pigments and phenolic compounds extracted from different microalgae species (e.g., *Chlorella vulgaris, Haematococcus pluvialis*) have already been studied for their antioxidant potential (Dardavila et al. 2023; Kepekçi & Saygideger 2012; Ruiz-Domínguez et al. 2019). Although there have been studies focused on optimizing phycocyanin extraction using organic solvent options and maceration (Silveira et al. 2007), there have been no reported optimization studies of the extraction of total pigments, total phenolic compounds and the antioxidant activity of the *Spirulina platensis* extracts. Thus, this study intends to shed light on the potential of NADES use in the extraction of valuable compounds, as a whole, from *Spirulina platensis* and their potential antioxidant activity.

**Fig. 4:**
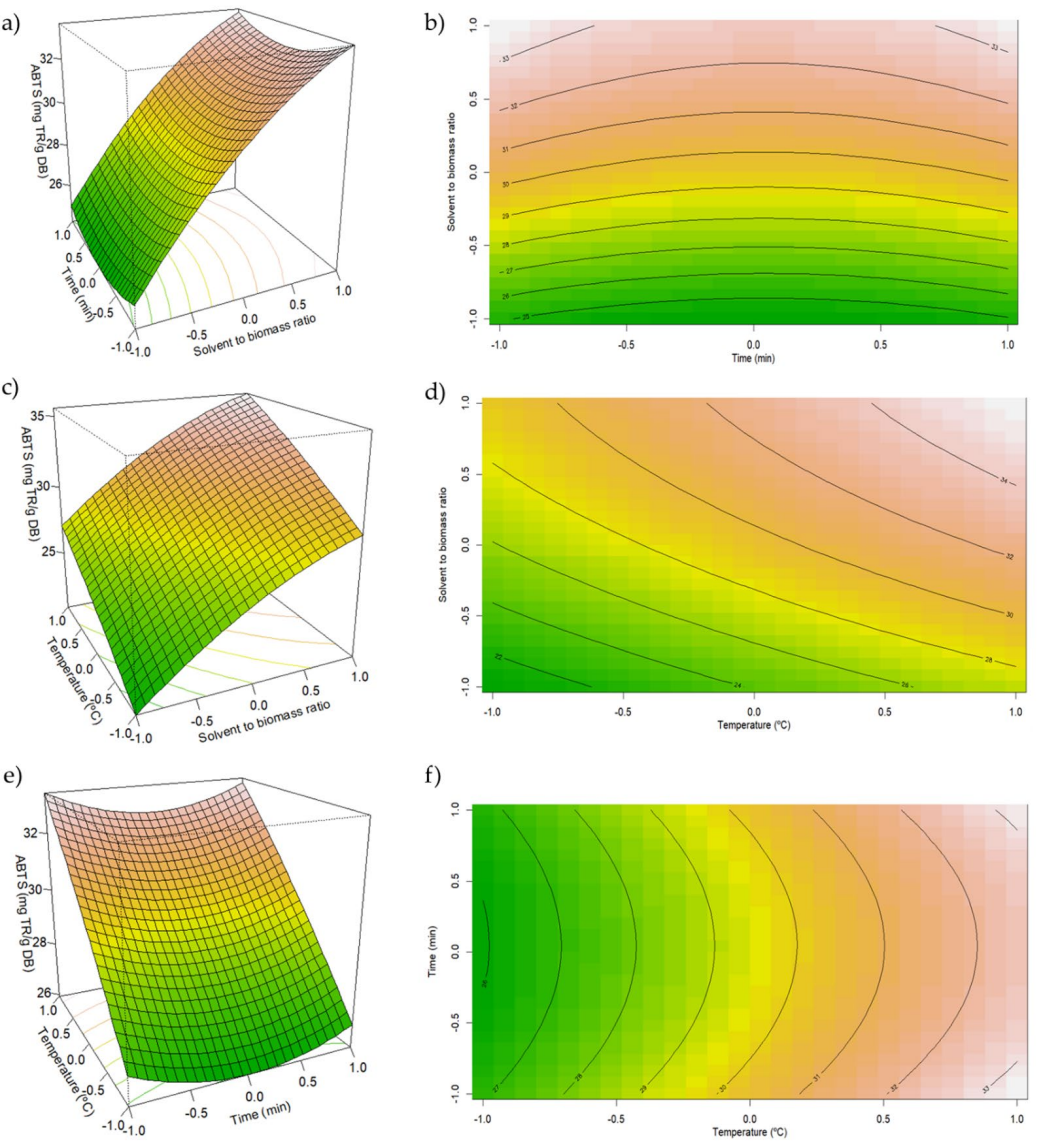
Response surface and contour plots representative of the interaction effects of (**a**, **b**) time and solvent-to-biomass ratio; (**c**, **d**) temperature and solvent-to-biomass ratio; (**e**, **f**) time and temperature on the antioxidant activity

Figure 4 illustrates the response surface and contour plots obtained in by RSM. From Fig. 4a, b, it can be drawn that a clear relationship between higher antioxidant activity with higher solvent-to-biomass ratio. In the study developed by Dardavila et al. (Dardavila et al. 2023), this relationship was also clear. Since the total pigment yield and TPC are clearly affected by the solvent-to-biomass ratio, where higher solvent-to-biomass ratios yields higher antioxidant compounds, it would be expected that a higher antioxidant activity would occur at the same solvent-to-biomass ratios.

From Fig. 4b, c, a clear indication of higher antioxidant activity with temperatures of 60 ℃ or higher is quite visible. From Fig. 4e, f, for a set solvent-to-biomass ratio, a clear indication of higher antioxidant activity with extraction temperature for a set time of extraction is also shown.

### Optimization of multiple response variables through desirability function analysis (DFA)

One of the primary objectives of the present study was to determine the optimal process parameters of the UAE (temperature, extraction cycle time, and solvent-to-biomass ratio), thus maximizing the total pigment yield, TPC, and antioxidant activities.

However, obtaining these responses under the same condition is difficult, since factors have distinct interest regions. In RSM optimization, two methodologies are most typically employed. The first approach is the superimposition of response contour plots and manual derivation of the desired value. Notwithstanding, Granato et al. (2010) assert that this graphical approach is inefficient and incapable of being automated. Therefore, the second strategy, desirability function analysis, was applied (Ahmed et al. 2022).

Desirability Function Analysis (DFA) is a prominent technique applied in academia and industrial arena for simultaneous optimization of multiple responses. The optimization process assigned desirability values ranging from 0 to 1 to each model's minimum and maximum responses (Devarajaiah & Muthumari 2018).

In DFA methodology, a process is unacceptable if any one of the responses is outside some desired limits. The objective is to obtain a set of optimum cutting conditions yielding most desirable individual response values. All the quality characteristics are converted to lie in [0 1], and individual desirability index is calculated. Weighted geometric mean of each pair of response variable is used to determine composite desirability index. Data sets with highest composite desirability are considered as optimum parameter settings that produce the most desirable quality characteristics under consideration.

**Fig. 5:**
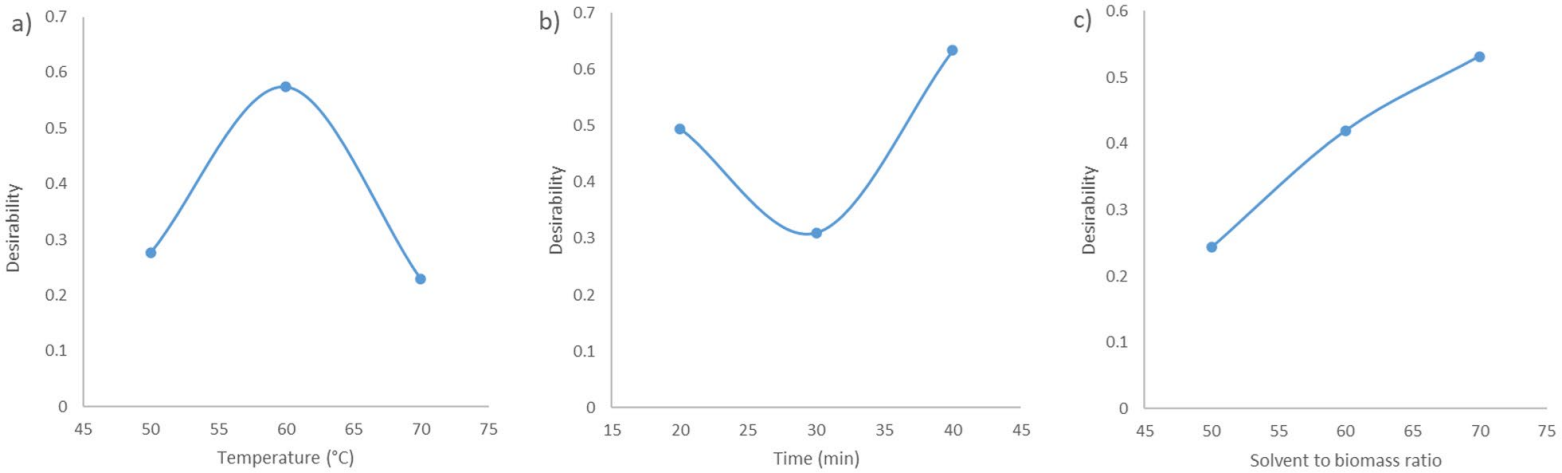
Desirability plots: **a** desirability for each temperature; **b** desirability for each extraction time; **c** desirability for each solvent-to-biomass ratio

Figure 5 represents the desirability plots for each independent variable used in this research work: temperature, time, and solvent-to-biomass ratio.

In Table 4, the determination of the individual desirability of the different experimental responses were determined according to Eq. (7), while the composite desirability that takes into account all of the individual desirability functions was determined using Eq. (8). After analyzing Table 4, it can be drawn that the experimental run number 11, showed the highest overall desirability out of all the experimental runs. Thus, the optimal experimental conditions are the following: temperature: 60 °C; extraction cycle time: 20 min; and solvent-to-biomass ratio: 70 mL/mg.

**Table 4:** Individual desirability and composite desirability of the different experimental responses

Run	Y1	Y2	Y3	d1	d2	d3	D
1	67.45	26.89	21.27	0.37	0.42	0.40	0.40
2	65.05	33.37	8.90	0.35	0.85	0.08	0.29
3	65.05	26.73	21.97	0.35	0.41	0.42	0.39
4	38.25	33.21	23.85	0.16	0.84	0.46	0.40
5	26.98	20.61	11.48	0.08	0.00	0.15	0.00
6	15.30	27.09	5.62	0.00	0.43	0.00	0.00
7	60.52	29.08	12.47	0.32	0.57	0.17	0.32
8	43.00	35.56	7.85	0.20	1.00	0.06	0.22
9	66.29	25.10	27.39	0.36	0.30	0.55	0.39
10	111.09	24.94	44.84	0.68	0.29	1.00	0.58
11	156.31	33.57	38.62	1.00	0.87	0.84	0.90
12	82.31	33.41	36.82	0.48	0.86	0.80	0.69
13	60.20	29.44	29.54	0.32	0.59	0.61	0.49
14	60.20	29.44	29.54	0.32	0.59	0.61	0.49
15	60.20	29.44	29.54	0.32	0.59	0.61	0.49

Y1: total pigment yield predicted by regression, Eq. 7; Y2: antioxidant activity predicted by regression, Eq. 8; Y3: total phenolic content (TPC) predicted by regression, Eq. 9; d(1–3): individual desirability for response variable Y(1–3); D: composite desirability;

After obtaining the optimal experimental conditions, three experimental runs were conducted at such conditions to evaluate the accuracy of the predictability of the models. Table 5 presents the predicted values for the total pigment yield, TPC and antioxidant activities and the experimental values obtained after the three experimental runs. Since the residual standard errors are low (Table 5), we can verify that the models are can accurately predict the optimal experimental conditions (extraction temperature, time of extraction, and solvent-to-biomass ratio) for the UAE of *Spirulina platensis* using a glucose/glycerol/water-based NADES.

**Table 5:** Experimental values of total pigment, TPC, and ABTS

Response variable	Predicted value at optimized conditions	Experimental values	Residual standard error (%)
Total pigment yield (mg/g DM)	156.31	165.19 ± 1.01	0.06
TPC (mg GAE/g DM)	33.57	36.50 ± 0.98	0.08
ABTS (mg TRE/g DM)	38.62	37.98 ± 0.58	0.02

## Conclusions

To the best of our knowledge, this is the first prospective study to optimize the extraction conditions of total pigments, total phenolics, and antioxidant activities from NADES/*Spirulina platensis* gel extract while using an NADES in the UAE. The present work looked to find an optimal extraction condition for total pigment yield, TPC, and antioxidant activity. RSM was successfully applied to optimize the extraction process and analyze the effects of extraction temperature, time, and solvent-to-biomass ratio and their interactions. From all the models generated using the RSM approach, only the TPC model demonstrated an adequate level of prediction accuracy (${R}^{2}$ > 0.9). After RSM, desirability functions were used to find the optimal conditions for the NADES/UAE extraction: temperature of 60 °C; extraction cycle time of 20 min, and a solvent-to-biomass ratio of 70 mL/mg. This was further validated through experimental runs. Furthermore, it is worth mentioning that it may be possible for the power, frequency, and duty cycle of ultrasound to affect the antioxidant activity and the TPC of the NADES/Spirulina gel-like extracts. Therefore, further studies should be conducted to understand the system behavior based on the aforementioned variables to improve and optimize extraction efficiency for industrial applications. Overall, this study should be considered a first step for the optimization of the extraction of antioxidative compounds derived from *Spirulina platensis*. Further research must be conducted towards scaling-up the process and evaluate the potential use of the resulting extracts in the materials, pharmaceutical, and packaging sectors.

## Data Availability

The authors declare that the data supporting the findings of this study are available within the article.

## Abbreviations

BBD: Box–Behnken design
CCD: Central composite design
DESs: Deep eutectic solvents
DFA: Desirability function analysis
DM: Dry matter
ILs: Ionic liquids
MAE: Microwave-assisted extraction
NADESs: Natural deep eutectic solvents
RSM: Response surface methodology
TPC: Total phenolic content
UAE: Ultrasound-assisted extraction
